# The evaluation of a composed program of continuing medical education for general practitioners

**Published:** 2014-07

**Authors:** MARZIEH MOATTARI, DAVOOD YADGARI, SEYED JALIL HOSEINI

**Affiliations:** 1 Quality improvement in Clinical Education Research Center, Shiraz University of Medical Sciences, Shiraz, Iran;; 2 Shalid Beheshtie University of Medical Sciences, Tehran, Iran

**Keywords:** Education, Evaluation, General practitioner

## Abstract

**Introduction**: The execution of composed educational programs for general practitioners is one of the most common methods of continuing medical education. This research project aims to evaluate one of these programs.

**Methods**: For this purpose, a pre- and post-test design was developed. The subjects consisted of 45 participants. They were tested in two stages: before and after the program. A questionnaire was also used to gather the participants’ views on four variables including teachers' behavior, the degree of achieving the objective of the program, objective of the learner, and satisfaction with the program.

**Results:** Based on the results of this study, the mean scores of the participants’ knowledge increased from 10.05 (pre test) to 12.61 (post test), (p<0.0001). In addition, the results showed that the teachers’ behavior and satisfaction with the program were rated by participants as the highest and least, respectively.

**Conclusion**: The results of this research are indicative of the effectiveness of the composed educational program in continuing medical education. Nevertheless, such programs are recommended to be further evaluated with more rigorous design.

## Introduction

Medical sciences change rapidly with new scientific information and technology (1); therefore, continuing education plays a vital role in presenting ever-growing knowledge, modern technology, and new orientation for the hearth care providers. To perform their professional responsibilities, physicians need to participate in continuing medical education (CME) (2). CME has been the focus of many studies conducted by medical teachers and health managers throughout the world. Convenience, Relevance, Individualization, Self-assessment, Interest and Speculation (CRISIS) were recommended to improve the effectiveness and quality of continuing medical education programs (3).

On the other hand, the quality control and establishment of effective continuing medical education programs have been under consideration from long ago and the need for their evaluation has repeatedly been cited (4-6). Nevertheless, the evaluation methods used have defects including lack of objectivity, repeatability, and feedback to learners and compatibility. Existing evidence suggests that CME programs in Iran have rarely been evaluated, and most of them end up only with an opinion poll. As a result, re-examination in the procedure and qualitative control of these programs is a well recognized requirement.

Comprehensive evaluation of CME programs needs sufficient time, energy and resource for planning and organizing evaluation. That's why the evaluation methods mostly focus on opinion evaluation. Although the effect of CME on behavior change has been investigated (7), there are some evidence of  investigating the knowledge and or opinions of the participants of CME program using question test before and after the  educational sessions (8), a questionnaire along with open discussion at the end of a symposium (6), a test and a questionnaire approving an increase in physician's self-confidence confronting with clinical and psychosocial symptoms (9), psychometric evaluation of participants taking part in an intensive 5-year continuing education program (10), and mailed questions on the topic of the educational package on the knowledge of etiology, diagnosis and the treatment of incontinency (11).

In an investigation of the status of CME of the Iranian Medical Society, it was concluded that in most CME programs complaints, violations and problems with medical documentation and the society’s culture are not considered. The main objection of the participants in the program was lack of harmony among the professional needs of the participants and clinical problems of the physicians with the subjects raised in the continuing educational programs (12). In another research project, the relationship between the subjects attending the continuing educational programs and health requirements and needs of the country was investigated. The researchers concluded that in most cases there is no harmony between the society’s needs and the length of time spent to teach and train the physicians in this regard (13).

Presently, CME is presented in most of the main universities and service providing centers of the country. This has been immensely expensive. Evidence suggests that in most cases, the evaluation of these programs is only conducted using the opinions of the participants which indeed cannot be illustrative of different aspects of evaluation (5). Considering the feasible methods of evaluation in the above-mentioned research projects, the present research aims at evaluating a training program held in Shiraz.

## Methods

In this pre-test post-test evaluative design study, 45 general practitioners who attended a training program participated. Five variables including “level of knowledge”, "meeting the program and participants' objectives", "teachers' behaviors", and "satisfaction with the program" were investigated using a knowledge test and a valid and reliable 19-item instrument for evaluation of continuing education program.

 The knowledge test was developed by the professors of Shiraz University of Medical Sciences. They were asked to develop relevant higher order multiple-choice questions in accordance with the objectives of their speech and present them to one of the researchers. The questions then were modified by an expert panel to ensure its validity. Also, the reliability of the test was measured using the result of the same program executed previously. KR20 of the test revealed a high reliability for the test (0.88). The same knowledge test was used in both pre-test and post-test.

To measure other variables, Henker and Hinshaw’s questionnaire was used. This questionnaire was developed based on Steven’s theoretical framework for measurement of the above-mentioned variable (14). The original questionnaire was translated into Persian by two bilingual experts. Two translations were compared and a consensus was made between the two translators. To measure the reliability of the questionnaire, it was applied in a similar CME course with a 2 two week interval. A test re-test reliability of 0.92 was obtained.

 At the beginning of the program, a brief description of the method in which the research was conducted was brought to the attention of the physicians. To make their identities confidential, researchers prepared numbered cards enclosing them in envelopes.  Each physician, then, selected an envelope (containing the card) and considered its number as his/her identity number. They were requested to write this card/number on their examination sheets and evaluation forms. The knowledge test was taken at the beginning of the first day and the end of the last day (the 4th day). The questionnaire for evaluation of other variables was distributed among the subjects at the end of the program. Every attempt was made to secure a good administration of the test, an appropriate environment, space and timing. Nevertheless, with respect to the inclusion of the codified number on the sheets (rather than the name, surname), the participants did not feel nervous or anxious at all.

To analyze the data, the paired t-test was used. For other data, multiple range test and ANOVA were used. Spss 14 (SPSS Inc, Chicago, IL, USA) was used and p<0.05 was considered as significant level 

**Ethical considerations**

Institutional Review Board (IRB) approval for the study was obtained from the Ethics Committee of the University (ECSUMS). Verbal consent was obtained from each physician. The purpose of study, voluntary participation, confidentiality and freedom to take the test and provide feedback on evaluation forms were reviewed. 

## Results

45 physicians participated in this study (10 females and 35 males) with a mean age of 32.5 ranging from 26 to 44. The participants' level of knowledge before and after the educational program as compared in the two administered test shows that the mean±standard deviation of the scores increased from 10.05±1.48 to 12.61±1.61, which is a significant difference (p<0.0001).

The mean scores of the items included in the opinion questionnaire showed that the mean scores given to the 19 existing items ranged from 3.91 to 5.27 (out of 6). Teachers' behaviors obtained the highest scores (Table 1). The lower points belonged to the following items:

- “Responsiveness of the program to actual problems encountered while working”
- “Appropriateness of the program organization,”
- “Ability of the program to fulfill expectations”
- “Comprehensiveness of the program”

Table 2 shows the results regarding the variables under consideration. Based on these findings, mean ratings given to the four variables was considerably favorable. But “Teachers’ behavior” obtained the highest mean and “Satisfaction with the program” the lowest one. The multiple range lest showed a significant difference between these two means.

**Table 1:** Participants’ mean rating of the items related to evaluation

Items outlined in the questionnaire	Mean ratings given by the participants		
	Mean±SD	Min	Max
Appropriateness of teachers’ behavior towards the participants as adult learners	5.27±0.65	4	6
Good knowledge of teachers	5.27±0.91	1	6
Difficulty of materials*	5.05±0.91	2	6
Degree to which the program is recommended to colleagues	4.84±0.86	3	6
Relevancy of the content to professional activities as a reason to participate	4.60±0.96	2	6
Relevancy of the materials to professional activities	4.57±0.85	3	6
Achieving the program objectives	4.57±0.95	3	6
Mismatch between personal objectives of taking part in the program and those of the program	4.56±1.08	2	6
Congruency between the content of the program and program objectives	4.53±0.88	2	6
Clarity of the program objectives at the beginning of the program	4.52±0.99	2	6
Match between the content of the program and professional activities	4.51±0.86	3	6
Usefulness of handouts and audio-visual materials	4.45±1.13	2	6
New viewpoints and values enforced by teachers	4.45±1.11	1	6
Not obtaining much from the program*	4.42±1.27	1	6
Incompatibility between the program objectives with their own objectives*	4.33±1.21	1.6	4.22
Responsiveness of the program to the actual problems encountered while working	4.22±1.00	1	6
Appropriateness of the program’s organization by teachers	4.07±1.02	2	6
Ability of the program to fulfill expectations	4.04±0.93	1	6
Comprehensiveness of the program	3.91±1.25	1	6

*These items were negatively scored

**Table 2:** Participants’ mean rating given to the related variables measured in the study

**Variables**	**Mean rating±SD**	**Confidence interval**
Teachers' behavior	4.75±0.60	4.56-4.93
Program objectives	4.51±0.63	4.31-4.71
Learner objectives	4.45±0.76	4.20-4.69
Satisfaction with the program	4.4±0.81	4.16-4.64

## Discussion

The result obtained from this evaluation indicates that participation in the continuing educational program is effective in enhancing the knowledge level of the physicians. Comparison of the mean rating obtained from the participants’ views on four other variables showed that the highest and lowest mean ranking belonged to the teachers' behavior and satisfaction with the program, respectively (14).

Although knowledge scores of the physicians were unfavorable at the pretest, they increased as the result of attendance in the composed CME program. In other studies, enhancing the knowledge level of physicians has been considered as evidence of the effectiveness of CME programs (8, 9, 15-17). It is clear that the increase in knowledge does not guarantee outcomes such as change in behavior/competency or performance and consequently a better care /service for the patients. However, we did not measure the changes in behaviour because expecting change in behavior is not plausible as the outcome of every CME program. Change in behavior can be influenced by many individual factors including learning styles, mental patterns, backgrounds, interests and personal experiences which are brought to the learning situation. Change in behavior, as an evidence of learning, may also be influenced by many other variables such as educational methods, personality, and environment. Lack of readiness to change is another factor associated with the inconsistency of behavioral change (18).

Extensive content and didactic teaching strategies usually applied in our CME programs are not appropriate for producing a change in behavior. It appears that the change in behavior would rather be expected after CME programs focusing on more specific contents. In such specific courses, medical records may be used as a tool for performance evaluation (19). Also didactic teaching strategies applied in our evaluated CME program provide few opportunities for group discussion. Such kinds of didactic sessions do not appear to be effective in changing the physicians’ performance. To expect change in behavior, professional practice, health care outcomes, and more interactive methods that enhance the participant’s activity and provide an opportunity to practice skills should be applied (20). Furthermore, educational activities such as community-based strategies, practice-based methods (reminders and patient-mediated strategies), and multiple interventions are shown to be most effective activities while audit and educational materials lead to a weaker outcomes (21). In one study, it was shown that complex interventions, such as the use of outreach visits or local opinion leaders, were effective in reducing the incidence of inappropriate performance (22). It is confirmed that broadly defined CME interventions using practice-enabling or reinforcing strategies consistently are effective in improving physicians’ performance and, in some instances, health care outcomes (23).

Therefore, complexities of the factors associated with the change in behavior make it difficult to attribute any change in behavior to a single CME program. Furthermore, comprehensive evaluation of CME programs is not feasible in all situations. Methodological and organizational barriers in conducting such evaluation programs have been highlighted as the reason for the scarcity of articles measuring the effect of continuing education programs on patients (24). Considering the limitation of our study, further research is needed to evaluate different aspects of the process and outcome of the program. Such type of comprehensive evaluation may be well reflected in the performance of physicians. In a proposed model called Cambridge Model, performance was identified as a product of competence, influences of the individual and impact of the system (25).

In the current study, we did not measure competence or performance of the physicians. However, considering the existing correlation between the scores of written tests and performance based tests (26) and the fact that medical knowledge test can predict actual clinical performance to the same extent as the multiple-station examination (27), we are hopeful that the increase in the knowledge level of those participating in the composed program would be effective in improving their performance.

The results of this research showed that items related to participants' expectations were rated at a moderately low level. This finding may be attributed to the lack of harmony between educational content and learners’ real needs, and/or program objectives. Lack of harmony between social needs and the time allotted to these needs during continuing education program in Iran was identified in previous research (13). This finding is very important and should be considered in our future CME planning. Furthermore, an evaluation process should be in place to ensure that the needs were addressed and met (28). “In one study it was shown that provision of CME based on the expressed needs of medical practitioners tends to be well received and highly valued by workshop respondents” (29).

A comparison of the mean rating of the participants’ views showed that teachers' behavior obtained the maximum mean rating. This result was not unexpected as very experienced teachers took charge of continuing education programs. This is not always the case. CME delivered through community-based educationally influential physicians is identified as an effective way to change the physicians’ behavior in small communities with no prior ongoing educational programs (30).

Although the mean ratings of the physicians' views were all in acceptable ranges, that for satisfaction with the program was lesser than the means of other variables. Satisfaction with the program, however, is subjective to subjectivity and might be affected by many individual factors such as motivation and interests. Also based on the findings of this evaluation, the following low rated items by our participants may explain the slightly lower rating of satisfaction with the program (31). These items are as follows: Responsiveness of the program to the actual problems encountered while working, Appropriateness of the program organization, by teachers, Ability of the program to fulfill expectations, and Comprehensiveness of the program. Our traditionally applied CME may result in more satisfaction if we base the program on the actual needs of the physicians as it was reported in other studies (32) and apply new interactive educational methods such as distance learning (31), videoconferencing (33) or bringing CME to the bedside (34) in our CME.

The evaluation of the CME program meets the criteria of the Accreditation Council for Continuing Medical Education (ACCME) which required the CME providers to measure the effectiveness of the CME activity in meeting the identified educational needs in terms of satisfaction, knowledge, or skills (35).

## Conclusion

In summary, this evaluation showed that participation in the form of CME program results in an increase in the knowledge level of physicians. Participants also rated the teachers' behavior as the highest rank. They achieved the program’s objectives and learning objectives and were moderately satisfied with the program. However, further evaluation using more rigorous design is suggested to delineate a better image of the effectiveness of CME programs.
